# Supplementary material for: Structural dynamics of IRE1 and its interaction with unfolded peptides
Source: eLife. 2026 Jul 23;14:RP106716. doi: 10.7554/eLife.106716 (PMC13395458; doi:10.7554/eLife.106716)
Supplement: Supplementary file 1. — All the peptides were simulated in complex with hIRE1α cLD dimer AlphaFold2 (AF2) model. All systems were solvated in either TIP3P or TIP4P-D water and with 0.15 M NaCl plus counterions. All simulation frames were saved with a time step of 0.25 ns. [file elife-106716-supp1.pdf]

Structural Dynamics of IRE1 and its Interaction with Unfolded Peptides

Supplementary File 1

System	water model	# atoms	box size (nm^3)	replicas x µs
hIRE1α cLD dimer (PDB)	TIP3P	232237	13.5^3	3 x 2 µs
hIRE1α cLD dimer (PDB)	TIP4P-D	317944	13.5^3	3 x 2 µs
hIRE1α cLD dimer (PDB initial)	TIP3P	205637	13.0^3	3 x 2 µs
hIRE1α cLD dimer (PDB initial)	TIP4P-D	283420	13.0^3	3 x 2 µs
hIRE1α cLD dimer (AF2)	TIP3P	205934	13.0^3	3 x 2 µs
hIRE1α cLD dimer (AF2)	TIP4P-D	283508	13.0^3	3 x 2 µs
hIRE1α cLD dimer (AF2) - DR1 loop	TIP3P	192369	12.7^3	3 x 0.5 µs
yeast Ire1 cLD dimer (PDB)	TIP3P	111743	10.6^3	3 x 2 µs
yeast Ire1 cLD dimer (PDB)	TIP4P-D	149414	10.6^3	3 x 2 µs
hIRE1α cLD dimer w/ valine8	TIP3P	205939	13.0^3	3 x 1 µs
hIRE1α cLD dimer w/ valine8	TIP4P-D	283519	13.0^3	3 x 1 µs
hIRE1α cLD dimer w/ MPZ1N (90°)	TIP3P	186946	12.6^3	3 x 1 µs
hIRE1α cLD dimer w/ MPZ1N (90°)	TIP4P-D	283529	13.0^3	3 x 1 µs
hIRE1α cLD dimer w/ MPZ1N (270°)	TIP3P	237572	13.6^3	3 x 1 µs
hIRE1α cLD dimer w/ MPZ1N (270°)	TIP4P-D	283553	13.0^3	3 x 1 µs
hIRE1α cLD dimer w/ MPZ1N (0°)	TIP3P	187024	12.6^3	3 x 1 µs
hIRE1α cLD dimer w/ MPZ1N (0°)	TIP4P-D	283541	13.0^3	3 x 1 µs
hIRE1α cLD dimer w/ MPZ1N-2X	TIP3P	205882	13.0^3	3 x 1 µs
hIRE1α cLD dimer w/ MPZ1N-2X	TIP4P-D	283497	13.0^3	3 x 1 µs
hIRE1α cLD dimer w/ MPZ1N-2X-RD	TIP3P	205709	13.0^3	3 x 1 µs
hIRE1α cLD dimer w/ MPZ1N-2X-RD	TIP4P-D	283401	13.0^3	3 x 1 µs
hIRE1α cLD dimer w/ MPZ1C	TIP3P	187078	12.6^3	3 x 1 µs
hIRE1α cLD dimer w/ MPZ1C	TIP4P-D	283542	13.0^3	3 x 1 µs
hIRE1α cLD dimer w/ 8ab1	TIP3P	187069	12.6^3	3 x 1 µs
hIRE1α cLD dimer w/ 8ab1	TIP4P-D	283523	13.0^3	3 x 1 µs
hIRE1α cLD dimer w/ OR-1	TIP3P	187079	12.6^3	3 x 1 µs
hIRE1α cLD dimer w/ OR-1	TIP4P-D	283536	13.0^3	3 x 1 µs
hIRE1α cLD dimer w/ V1rb2-1	TIP3P	187039	12.6^3	3 x 1 µs
hIRE1α cLD dimer w/ V1rb2-1	TIP4P-D	283475	13.0^3	3 x 1 µs
hIRE1α cLD dimer w/ V1rb2-2	TIP3P	187013	12.6^3	3 x 1 µs
hIRE1α cLD dimer w/ V1rb2-2	TIP4P-D	283462	13.0^3	3 x 1 µs
hIRE1α cLD dimer Y161R w/ MPZ1N-2X	TIP3P	205910	13.0^3	3 x 1 µs
hIRE1α cLD dimer Y161R w/ MPZ1N-2X	TIP4P-D	283529	13.0^3	3 x 1 µs
hIRE1α cLD dimer E102R w/ MPZ1N-2X	TIP3P	192413	12.7^3	3 x 1 µs
hIRE1α cLD dimer E102R w/ MPZ1N-2X	TIP4P-D	263821	12.7^3	3 x 1 µs
hIRE1α cLD dimer x2 w/ MPZ1N-2X	TIP4P-D	802187	20.7^3	1 x 0.2 µs
hIRE1α cLD monomer w/ BiP	TIP3P	401856	16.2^3	3 x 1 µs
hIRE1α cLD monomer w/ BiP-ADP	TIP3P	324700	15.1^3	3 x 1 µs
hIRE1α cLD monomer w/ BiP-ATP	TIP3P	385264	16.1^3	3 x 1 µs

The simulations performed in this work are summarised here. All the peptides were simulated in complex with hIRE1α cLD dimer AlphaFold2 (AF2) model. All systems were solvated in either TIP3P or TIP4P-D water and with 0.15 M NaCl plus counterions. All simulation frames were saved with a timestep of 0.25 ns.
