# Supplementary material for: Structural dynamics of IRE1 and its interaction with unfolded peptides
Source: eLife. 2026 Jul 23;14:RP106716. doi: 10.7554/eLife.106716 (PMC13395458; doi:10.7554/eLife.106716)
Supplement: Supplementary file 2. [file elife-106716-supp2.pdf]

Structural Dynamics of IRE1 and its Interaction with Unfolded Peptides
Supplementary File 2

Polypeptide	$K_{1/2}$ [μM]
MPZ1-N	16
MPZ1N-2X	0.456
MPZ1-C	572
MPZ1N-2X-RD	binding impaired
8ab1	5

Available binding affinities ($K_{1/2}$) of the unfolded polypeptides studied to hIRE1α [1, 2].

References

[1] Elif Karagöz G, Acosta-Alvear D, Nguyen HT, Lee CP, Chu F, Walter P. An unfolded protein-induced conformational switch activates mammalian IRE1. eLife. 2017;6:e30700.

[2] Kettel P, Marosits L, Spinetti E, Rechberger M, Giannini C, Radler P, et al. Disordered regions in the IRE1 ER lumenal domain mediate its stress-induced clustering. The EMBO Journal. 2024 Oct;43(20):4668-98.
