# Supplementary material for: Structural dynamics of IRE1 and its interaction with unfolded peptides
Source: eLife. 2026 Jul 23;14:RP106716. doi: 10.7554/eLife.106716 (PMC13395458; doi:10.7554/eLife.106716)
Supplement: Supplementary file 3. [file elife-106716-supp3.pdf]

# Structural Dynamics of IRE1 and its Interaction with Unfolded Peptides

# **Supplementary File 3**

| | hIRE1$\alpha$ LD wild-type | hIRE1$\alpha$ LD E102R | hIRE1$\alpha$ LD Y161R |
|---|---|---|---|
| $K_D$ [µM] | 2.14 | 6.35 | 5.433 |
| $K_D$ 95% confidence interval [µM] | 1.809 to 2.528 | 5.835 to 6.959 | 4.296 to 7.095 |
| $R^2$ | 0.9918 | 0.9992 | 0.9851 |

Goodness-of-fit parameters of binding curves of MPZ1-N-2X peptide to hIRE1$\alpha$ LD for fluorescence anisotropy experiments.
