# Supplementary material for: Structural dynamics of IRE1 and its interaction with unfolded peptides
Source: eLife. 2026 Jul 23;14:RP106716. doi: 10.7554/eLife.106716 (PMC13395458; doi:10.7554/eLife.106716)
Supplement: Peer review history

# Editor report: eLife Assessment

**DOI:** 10.7554/eLife.106716.3.sa0

**Authors:** Qiang Cui

**Affiliations:** Boston University United States

**Keywords:** Convincing, Solid, Important

In this **important** study, the authors conducted atomistic molecular dynamics simulations to probe the interactions between IRE and unfolded peptides. The results help reconcile contradicting experimental findings in the literature and offer mechanistic insights into the activation of the unfolded protein response. The atomistic molecular dynamics simulations performed are **solid**, leading to **convincing** conclusions that are partly supported by experimental validations. The use of unbiased molecular dynamics simulations, while appropriate for the current system due to its complexity, limits the time scale of events that can be observed and therefore the proposed mechanism of recognition merits further confirmation by future studies.

# Referee report: Reviewer #1 (Public review)

**DOI:** 10.7554/eLife.106716.3.sa1

Summary:

This work provides structural and mechanistic insights into the disordered protein recognition process inside the endoplasmic reticulum by the inositol-requiring enzyme 1. Using state-of-the-art molecular dynamics simulation tools, the authors propose a mechanism of disordered protein recognition that reconciles contradictory findings of biochemical and structural biology experiments.

Strengths:

(1) All MD simulations have been carried out in triplicates, and several different folded conformations were generated using alphafold2. This provides adequate statistics to draw meaningful conclusions from the simulations.

(2) Potential limitations of the disordered protein force fields and water models have been taken into consideration. Particularly, performing the simulation in both TIP3P and TIP4PD water models ensures that the conclusions drawn are not influenced by the force field choice.

(3) The binding of a large number of disordered peptides was investigated, ensuring that the conclusions drawn about disordered peptide recognition are sufficiently general.

Weaknesses:

(1) The timescales of the peptide recognition and unbinding process are much longer than what can be sampled from unbiased simulations. Therefore, the proposed mechanism of recognition should only be considered a hypothesis based on the results presented here. For example, peptides that do not dissociate within one microsecond MD simulation are considered to be stable binders. However, they may not have a viable way to bind to the narrow protein cleft in the first place.

(2) Oftentimes, representative structures sampled from MD simulation are used to draw conclusions (e.g., Figure 4 about the role of R161 mutation in binding affinity). This is not appropriate as one unbinding event being observed or not observed in a microsecond-long trajectory does not provide sufficient information about the binding strength of free energy difference.

Comments on revisions:

The authors have adequately addressed my comments. I have no further comments.

# Referee report: Reviewer #2 (Public review)

**DOI:** 10.7554/eLife.106716.3.sa2

Summary:

In this manuscript, the authors investigated the interactions between IRE and unfolded peptides using all-atom molecular dynamics simulations. The interactions between a couple of unfolded peptides and IRE provide mechanistic insight on the activation of the UPR.

Strengths:

- Well-written manuscript accessible for a broad biological audience

- State-of-art structural predictions and all-atom simulations

- Validation with existing experimental data

- Clear schematic diagram summarizing mechanisms learned from simulations

- Error estimate included

- Shared simulation data and code in public repository

Weakness:

No major concerns remain after revision.

Comments on revisions:

The authors have addressed all my questions from the previous assessment. I do not have more suggestions.

# Referee report: Reviewer #3 (Public review)

**DOI:** 10.7554/eLife.106716.3.sa3

Summary:

In this important work, the authors use extensive MD simulations to study how the IRE1 protein can detect unfolded peptides. Their study consolidates contradictory experimental results and offers a unique view of the different sensing models proposed in the literature. Overall, it is an excellent study that is quite extensive. The research is solid, meticulous, and carefully performed, leading to convincing conclusions.

Strengths:

The strength of this work is the extensive and meticulous molecular dynamics simulations. The authors use and investigate different structural models, for example carefully comparing a model based a PDB structure with reconstructed loops with a AlphaFold 2 Multimer model. The authors also investigate a wide range of different protein structural models that probe different aspects of the peptide-sensing process. Additionally, the authors experimentally validate a part of the simulation results. These solid and meticulous MD simulations allow the authors to obtain convincing conclusions concerning the peptide-sensing process of the IRE1 protein.

Weaknesses:

A potential weakness of the study is the use of equilibrium (unbiased) molecular dynamics simulations, which means only processes and conformational changes on the microsecond timescale can be probed. Furthermore, there can be inaccuracies and biases in the description of unfolded peptides and protein segments due to the protein force fields. Here, it should be noted that the authors do acknowledge these possible limitations of their study in the conclusions. Furthermore, in the revised version, the authors partly address this weakness by employing orthogonal simulation methods and experimental techniques.

Comments on revisions:

The authors have addressed all the issues that I raised in my previous report.

# Author comment: Author response

**DOI:** 10.7554/eLife.106716.3.sa4

**Authors:** Elena Spinetti, Grzegorz Ścibisz, Gülsün Elif Karagöz, Roberto Covino

**Affiliations:** Goethe University Frankfurt and Frankfurt Institute for Advanced Studies Frankfurt am Main Germany; Max Perutz Laboratories Vienna Austria; Max Perutz Laboratories Vienna Austria; Goethe University Frankfurt Frankfurt am Main Germany

The following is the authors’ response to the original reviews

**Public Reviews:**

**Reviewer 1 (Public review):**

(1) "The timescales of the peptide recognition and unbinding process are much longer than what can be sampled from unbiased simulations. Therefore, the proposed mechanism of recognition should only be considered a hypothesis based on the results presented here. For example, peptides that do not dissociate within one one-microsecond MD simulation are considered to be stable binders. However, they may not have a viable way to bind to the narrow protein cleft in the first place."

We thank the Reviewer for this valuable feedback and we agree with the Reviewer. Our work on the IRE1 cLD activation mechanism is focused on generating a hypothesis of the binding mechanism driven by MD simulations. We recognize the limitations in defining a stable binder due to the time scales sampled. However, our primary focus was to sample and characterize a possible binding pose in the center of the cLD dimer. We contextualized our statements about stable binders and limited our claims to stating that the protein-peptide complex is stable within 1 µs-long simulations. However, we believe that our finding that the cLD dimer groove is not able to accommodate peptides is solid, as the steric impediment described is present in all our replicas, both with and without peptides, in a cumulative sampling time of 24 µs without peptides and 66 µs with peptides. Additionally, we included a plot showing the distribution of groove width across all replicas.

Addition to the text. (Results section: Unfolded polypeptides bind to hIRE1*α* cLD dimer surface) The title was changed from “Unfolded polypeptides can stably bind to hIRE1*α* cLD dimer” to “Unfolded polypeptides bind to hIRE1*α* cLD dimer surface”

Addition to the text. (Figure 15 A legend) “(A) Distributions of the groove width of peptide-bound cLD dimers throughout all simulations performed. The left column shows the values for the three replicas in TIP3P water, while the right column displays those for the three replicas in TIP4P-D water.”

(2) Oftentimes, representative structures sampled from MD simulation are used to draw conclusions (e.g., Figure 4 about the role of R161 mutation in binding affinity). This is not appropriate as one unbinding event being observed or not observed in a microsecond-long trajectory does not provide sufficient information about the binding strength of the free energy difference.

We thank the Reviewer for the insightful comment. As explained in the previous point, we believe that our simulations provide useful hypotheses. We are aware of the limitations due to the timescale and agree that these limitations cannot be overcome with standard equilibrium simulations. To address these limitations, used orthogonal methods, specifically MM/PB(GB)SA calculations, to calculate binding free energies from existing trajectories. We added predictions of all the peptides using AlphaFold 3, to confirm the binding region. Importantly, we now provide experimental results to assess the binding affinity of cLD dimer mutants E102R and Y161R.

Addition to the text. (Results section: Unfolded polypeptides bind to hIRE1*α* cLD dimer surface) “AlphaFold3 predictions of the complexes indicate that the peptides adopt the same preferred orientation, despite being predominantly helical (Supplementary Fig. 16A). We further assessed the MPZ-derived peptide complexes using MM/PBSA free energy calculations over the final 250 ns of each simulation replica (see Methods), finding binding enthalpies consistent with our observations (Supplementary Fig. 16B). In particular, MPZ1N-2X exhibited the lowest binding energy, whereas MPZ1N-2X-RD showed the highest.”

Addition to the text. (Figure 16 legend) “(A) Prediction of AlphaFold 3 for hIRE1*α* cLD dimer in complex with peptides. Colors represent the confidence of the prediction (plDDT). (B) Difference in enthalpy (enthalpy of binding, *∆H*) as an estimate of the binding free energies of unfolded polypeptides to hIRE1α cLD dimer derived from MM/PBSA calculations of our peptide simulations.”

Addition to the text. (Figure 4 G legend) “(G) Fluorescence anisotropy measurements of labeled MPZ1N-2X binding to hIRE1*α* LD wild type and mutants E102R and Y161R.”

Addition to the text. (Results section: Point mutations destabilize unfolded peptide binding to cLD) “To experimentally test whether these residues are involved in hIRE1α LD’s interaction with peptides, we expressed and purified these mutants and conducted fluorescence anisotropy experiments using fluorescently labeled MPZ1N-2X peptide. We could purify both E102R and Y161R mutants to high purity (Supplementary Fig. 18C). They both behaved similarly to the wild type during purification. Notably, both E102R and Y161R mutants demonstrated around two-fold lower binding affinity (Fig. 4G, E102 *K*_1/2_ = 6.35 µM and Y161R *K*_1/2_ = 5.4 µM, Supplementary Table 3) compared to the wildtype (*K*_1/2_ = 2.14 µM, Supplementary Table 3), revealing that the protein’s central area is crucial for binding unfolded proteins and that binding activity occurs within the pocket defined by E102 and Y161.”

Addition to the text. (Figure 4G legend) “(G) Fluorescence anisotropy measurements of labeled MPZ1N-2X binding to hIRE1*α* LD wild type and mutants E102R and Y161R.”

Addition to the text. (Supplementary Table 3)

**Reviewer 2 (Public review):**

(1) Improving presentation to include more computational details.

We thank the Reviewer for raising this critical point. We agree that the manuscript is tailored for a biology audience, as the data are particularly relevant for that community. Nevertheless, we also understand the importance of providing sufficient methodological detail for computational readers. We added more references to the methods for computational information in the main text.

(2) More quantitative analysis in addition to visual structures.

We added an uncertainty estimate for the HDX calculations using bootstrapping and included additional information on bond distances for E102 and Y161. We also incorporated time-series data showing the distance of the peptide from the groove across all replicas.

Addition to the text. (Figure 1C legend) “(C) The deuterated fraction obtained from experimental results (dashed line, shaded area indicates the error we calculated from bootstrapping) published by Amin-Wetzel et al. and the fraction computed from MD simulations (solid lines, blue for TIP3P water and orange for TIP4PD water) for the PDB and AF model at incubation time point 0.5 min. This time point corresponds to experimental incubation times, not MD simulation time. Each point represents the mean value derived from three replicas and two monomers per replica. The error bars were obtained from bootstrapping. Below each absolute value plot, we report the discrepancy, which is defined as the difference between the simulated and experimental deuterated fractions, with the shaded area indicating the corresponding error.”

Addition to the text. (Figure 15B legend) “(B) Minimum groove-peptide distance over time for all simulations of cLD dimer in complex with a peptide. The left column shows the values for the three replicas in TIP3P water, while the right column displays those for the three replicas in TIP4P-D water.”

**Reviewer 3 (Public review):**

A potential weakness of the study is the usage of equilibrium (unbiased) molecular dynamics simulations, so that processes and conformational changes on the microsecond time scale can be probed. Furthermore, there can be inaccuracies and biases in the description of unfolded peptides and protein segments due to the protein force fields. Here, it should be noted that the authors do acknowledge these possible limitations of their study in the conclusions.

We appreciate the Reviewer’s thoughtful comment. As noted in our response to Reviewer 1, we addressed the concern about sampling by applying orthogonal methods and experimental techniques. We agree with the Reviewer that some form of enhanced sampling is necessary if we want to assess binding in a more quantitative way, e.g., via free energy calculations. However, we also realize that applying any enhanced sampling scheme to our system is very challenging, given its large size and the complex peptide-protein interactions, which are not easily captured in a few collective variables. After a careful assessment and some preliminary tests, we decided that estimating free energies using enhanced sampling would necessitate a separate paper due to both the conceptual complexity of the project and the size of the necessary sampling campaign.

**Recommendations for the authors:**

**Reviewer #1 (Recommendations for the authors):**

(1) Some enhanced sampling or path sampling simulations may be carried out to identify the peptides’ binding and unbinding mechanisms to the protein. This can show whether the disordered peptides studied in this work do indeed bind to the protein.

We thank the Reviewer for this constructive criticism. We acknowledge the limitations associated with investigating binding and unbinding mechanisms of disordered peptides within the time scales accessible to our equilibrium simulations. However, the primary objective of our study was to sample and characterize a plausible binding pose at the center of the cLD dimer. We wanted to understand if unfolded model peptides require an open groove able to contain them to bind to IRE1’s core luminal domain or if binding also in the absence of an open groove.

Enhanced sampling is, of course, an important strategy to overcome the limits of equilibrium simulations. However, we note that implementing enhanced sampling approaches in this system poses significant challenges due to its large size and the complexity of peptide–protein interactions, which cannot be easily captured using a limited set of collective variables. We decided that a thorough application of enhanced sampling would therefore constitute a separate study. Instead, we decided to validate our simulations in two ways: (1) we ran a new set of free energy calculations, and (2) we tested key predictions in experiments, adding significant new data to strengthen the conclusions of our manuscript.

To evaluate whether the binding free energies of MPZ-derived peptides to human IRE1*α* cLD dimers are consistent with experimentally reported binding constants, we employed the MM/PBSA (Molecular Mechanics/Poisson–Boltzmann Surface Area) method. Calculations were performed over the final 250 ns of each simulation replica using the Single Trajectory Protocol (STP), which avoids the need for additional simulations. This approach provides an estimate of the effective binding free energy (i.e., enthalpy of binding) by accounting for bonded and non-bonded interactions, as well as solvation contributions. The entropic contribution, being computationally more demanding and subject to additional approximations, was not included. Binding enthalpies were obtained for MPZ1-N (in different initial orientations), MPZ1-C, MPZ1-N-2X, and MPZ1-N-2X-RD. The results indicated small differences in effective binding energies between the shorter peptides (MPZ1-N and MPZ1-C), whereas MPZ1-N-2X exhibited the lowest binding energy and MPZ1-N-2X-RD the highest, consistent with experimental trends. These findings support the reliability of our model and sampling strategy as a framework for analyzing peptide binding conformations to cLD.

We identified residues E102 and Y161 as key contributors to the binding of unfolded peptides in our simulations. Contact analysis revealed these residues as binding hotspots, centrally located within the observed interaction regions. To probe their relevance, we conducted simulations of cLD dimers with single arginine mutations in these residues, aimed at disrupting these hotspots through charge repulsion. These simulations revealed increased instability of the MPZ1N2X on the cLD dimer surface. We further validated these findings experimentally using fluorescence anisotropy assays. Fluorescently labeled MPZ1N-2X was titrated with purified cLD mutants (E102R and Y161R), and anisotropy measurements were fitted to derive *K*_1/2_ values. Both mutations resulted in approximately a two-fold reduction in binding affinity relative to the wild-type cLD, confirming the importance of these residues in stabilizing peptide binding.

Addition to the text. (Results section title: Unfolded polypeptides bind to hIRE1*α* cLD dimer surface) “We further assessed the MPZ-derived peptide complexes using MM/PBSA free energy calculations over the final 250 ns of each simulation replica (see Methods), finding binding enthalpies consistent with our observations (Supplementary Fig. 16B). In particular, MPZ1N-2X exhibited the lowest binding energy, whereas MPZ1N-2X-RD showed the highest.”

Addition to the text. (Results section title: Unfolded polypeptides bind to hIRE1α cLD dimer surface) “Thus, we investigated how the point mutations of two key residues, E102R and Y161R, would affect peptide binding by simulating the cLD mutant in complex with MPZ1N-2X (Fig. 4C-E). We initialized the systems in the pose described for the other peptide-cLD systems described earlier (Fig. 3B, t = 0 µs). In simulations of the wild-type (WT) cLD dimer, the peptide generally remained near the center (Fig. 4C,F). By contrast, MPZ1N-2X displayed reduced binding to E102R, fully dissociating in one TIP4P-D replica (Fig. 4E,F). A similar trend was observed for Y161R, where one partial dissociation event occurred (Fig. 4D,F). Comparative analysis of MPZ1N-2X contact sites on the WT and mutant cLD dimers (Supplementary Fig. 17B-D) revealed that, in the presence of mutations, the peptide engages a broader surface region rather than remaining centrally localized, while forming fewer contacts with the specific residues (Supplementary Fig. 18A-B).”

Addition to the text. (Results section title: Unfolded polypeptides bind to hIRE1*α* cLD dimer surface) “To experimentally test whether these residues are involved in hIRE1α LD’s interaction with peptides, we expressed and purified these mutants and conducted fluorescence anisotropy experiments using fluorescently labeled MPZ1N-2X peptide. We could purify both E102R and Y161R mutants to high purity (Supplementary Fig. 18C). They both behaved similarly to the wild type during purification. Notably, both E102R and Y161R mutants demonstrated around two-fold lower binding affinity (Fig. 4G, E102 *K*_1/2_ = 6.35 µM and Y161R *K*_1/2_ = 5.4 µM, Supplementary Table 1) compared to the wildtype (*K*_1/2_ = 2.14 µM, Supplementary Table 1), revealing that the protein’s central area is crucial for binding unfolded proteins and that binding activity occurs within the pocket defined by E102 and Y161.”

Addition to the text. (Figure 4 legend) “(E) Side view snapshot after 1 µs of simulation of E102R hIRE1*α* cLD dimer (gray) in complex with MPZ1N-2X (orange). The amino acid R102 on both monomers is represented in magenta sticks. (F) Time series of the minimum groove-peptide distance for MPZ1N-2X simulated in complex with wild-type, E102R, and Y161R hIRE1*α* cLD dimer in TIP3P (3 replicas) and TIP4P-D (3 replicas) water. The darker lines show the rolling average over 25 frames, while the shaded lines represent the raw data. (G) Fluorescence anisotropy measurements of labeled MPZ1N-2X binding to hIRE1*α* LD wild type and mutants E102R and Y161R.”

Addition to the text. (Methods section: Binding free energy calculations (MM/PBSA)) “The binding free energy of noncovalently bound complexes of human IRE1 cLD and peptides was calculated with MM/PBSA (Molecular mechanics/PoissonBoltzmann Surface Area) method via gmx_MMPBSA (version 1.6.4)[1, 2]. The Poisson-Boltzmann method was used to estimate the electrostatic contribution to solvation free energy as recommended for data obtained with the CHARMM force field. The contribution of the entropic term was omitted, obtaining effective binding free energy values, or enthalpy of binding (∆*H*). We used the Single Trajectory Protocol (STP), using the cLD-peptide simulations as input. The calculations were performed on the last 250 ns of each replica. Single-term total non-polar solvation free energy (*inp* = 1) was used. The charmm_radii (*PBRadii* = 7) was used to build amber topology files [3]. The default parameters were applied for other terms.”

Addition to the text. (Methods section: Protein purification) “To express hIRE1*α* LD (24-443) human cDNA sequences were cloned into pET47b(+) to create a coding sequence with N-terminal His6-tag. Mutations of hIRE1*α* LD were introduced by overlap extension PCR and restriction cloning into pET47b(+). For expression of the proteins, the plasmid of interest was transformed into *Escherichia coli* strain BL21DE3* RIPL (Agilent Technologies). Cells were grown in Luria Broth until OD600=0.6-0.8. Protein expression was induced with 0.6 mM IPTG, and cells were grown in 20°C overnight. For purification, cells after harvesting were resuspended in Lysis Buffer (50 mM HEPES pH 7.2, 400 mM NaCl, 20 mM imidazole, 5% glycerol, 5 mM *β*-mercaptoethanol) and were lysed in Constans Systems cell disruptor at 25 000 psi. The supernatant was collected after centrifugation for 45 minutes at 48000×g in 4°C. Supernatant was loaded onto Ni-NTA column (Cytiva) and the protein eluted with a linear gradient of imidazole from 20 to 500 mM. Fractions containing the protein were diluted 1:8 with anion exchange wash buffer (50 mM HEPES pH 7.2, 5 mM *β*-mercaptoethanol), loaded onto HiTRAP-Q ion exchange column (Cytiva) and eluted with a linear gradient from 50 mM to 1 M NaCl. Afterwards, the His6tag was removed by cleavage with Precission protease (GE Healthcare, 1 µg of enzyme per 100 µg of protein). The cleavage was performed overnight in 4°C. The protein sample after cleavage was loaded onto a Ni-NTA column, and the flow-through containing protein without the tag was collected. The protein was further purified on a Superdex 200 10/300 gel filtration column equilibrated with Buffer A (25 mM HEPES pH 7.2, 150 mM NaCl, 2 mM DTT). Protein concentrations were determined using extinction coefficient at 280 nm predicted by the Expasy ProtParam tool (http://web.expasy.org/protparam/).”

Addition to the text. (Methods section: Fluorescence anisotropy) “For fluorescence anisotropy measurements, the MPZ1-N-2X peptide attached to 5 carboxyfluorescein (5-FAM) at its N-terminus was obtained from GenScript at >95% purity. Binding affinities of hIRE1*α* LD mutants to FAM-labeled peptides were determined by measuring the change in fluorescence anisotropy on a Tecan CM Spark Micro Plate Reader with excitation at 485 nm and emission at 525 nm with increasing concentrations of hIRE1*α* LD variants. Measurements were performed in Buffer A supplemented with Tween 20 (25 mM HEPES pH 7.2, 150 mM NaCl, 2 mM DTT, 0.025% Tween 20). Fluorescently labeled peptides were used in a concentration of 90 nM. The reaction volume of each data point was 25 *µ*L and the measurements were performed in 384-well, black flat-bottomed plates (Corning) after incubation of peptide with hIRE1*α* LD variants for 30 min at 25◦C. Binding curves were fitted using Prism Software (GraphPad) using the following equation: *F_bound_* = *r_free_* +(*r_max_* − *r_free_*)/(1+10((Log *K*_1/2_ −x)·*n_H_*)), where *F_bound_* is the fraction of peptide bound, *r_max_* and *r_free_* are the anisotropy values at maximum and minimum plateaus, respectively. *n_H_* is the Hill coefficient and x is the concentration of the protein in log scale. Curve-fitting was performed with minimal constraints to obtain *K*_1/2_ values with high *R*^2^ values. However, as this equation does not consider the equilibria between hIRE1*α* LD dimers/oligomers, these apparent *K*_1/2_ values do not reflect the dissociation constant.”

(2) Wherever possible, conclusions related to binding affinity should not be drawn from single unbinding events. For example, the title of Figure 4, "Single point mutation of cLD alters the binding affinity of unfolded peptide," should be softened. Similar changes should be made throughout the manuscript where such claims have been presented.

We thank the Reviewer for highlighting this important point. In the revised manuscript, we have adjusted the text to remove or soften conclusions related to binding affinity that were based on single unbinding events in the MD simulations.

Addition to the text. (Figure 4 title) “Single point mutations of cLD alter the binding of unfolded peptide MPZ1N-2X.”

Addition to the text. (Results section title: Unfolded polypeptides can stably bind to hIRE1*α* cLD dimer) “Unfolded polypeptides bind to hIRE1*α* cLD dimer surface.”

Addition to the text. (Results section: Unfolded polypeptides bind to hIRE1*α*α cLD dimer surface) “Our goal was to elucidate a potential binding pose and identify the relevant features of unfolded proteins and the cLD that affect the binding.”

**Reviewer #2 (Recommendations for the authors):**

(1) A table of all simulated trajectories, including simulation conditions, number of replicas, box size, number of atoms, equilibration length, recording time step, number of frames for further analysis.

We thank the Reviewer for this helpful suggestion. We have added a summary table of all simulations, including the requested details, to the Supplementary Information (Table 1).

Addition to the text. (Supplementary figures and tables: Table 2)

(2) The current NVT equilibration time was 0.125ns, and then no productive NPT simulations were mentioned as equilibration. Even though this is a simulation of mostly folded structures, it still takes some time for these amino acids to relax within the force field.

We thank the Reviewer for this constructive comment and acknowledge the validity of the concern. However, our simulations were extensively sampled, and equilibration was achieved within the first 50 ns of the production runs. Therefore, the segments of the trajectories from which we draw conclusions correspond to equilibrated states (see RMSD analysis, Figure 1). Additionally, binding free energy calculations (MM/PBSA) were carried out on the last 250 ns of the simulation replicas.

(3) At least three histograms were presented in Figure 2C, which I guess is from multiple simulations, and does not seem to be discussed.

We thank the Reviewer for pointing out the lack of reference to Figure 2C. We added the correct reference to the text where the groove width of luminal domains of human and yeast is discussed.

**Author response image 1.** RMSD analysis of human IRE1_α_ cLD dimer simulated in complex with unfolded peptides.

Addition to the text. (Results section: The putative groove of human IRE*α* cLD is dynamic but unable to contain peptides) In simulations of the dimeric structures, the average groove width was 7.3 ± 0.1 Å for the human cLD and 8.9 ± 0.1 Å for the yeast cLD, averaged over three TIP3P and three TIP4P-D replicas per system (Fig. 2C).

(4) The comment regarding the CHARMM force field on Page 6 is not justified. Actually the force field the authors used (CHARMM36m, Jing et al Nat Methods 2016) did include scaling of TIP3P LJ parameters to correctly capture the dimensions of the intrinsically disordered proteins (IDPs). However, the authors cited a couple of examples of literature of previous versions of CHARMM force fields and commented that it cannot capture IDP dimensions with TIP3P.

We thank the Reviewer for pointing out this source of confusion. We cited the main papers of CHARMM as [4, 5], which were misleading, and following the Reviewer’s advice, we removed these citations.

Addition to the text. (Results section: The hIRE1*α* cLD forms a stable dimer) “Current all-atom force fields used in MD simulations are mainly designed to reproduce the dynamics of folded and globular proteins [6].”

(5) I am fine that the authors used TIP4PD with CHARMM36m, but caution should be taken for such a combination of protein and water force fields. Note that when optimizing force fields for IDPs, one often has to balance protein-water interactions by either enhancing protein-water interactions, enhancing water dispersions, or reducing protein-protein interactions. So, all such optimization is dependent on both protein and water force fields. TIP4PD was designed to pair with Amber99sb-ildn or, most recently, Amber99sb-disp instead of CHARMM36m. This could result in rescaling of LJ parameters.

We thank the Reviewer for raising this issue. We argue that the TIP4P-D water model has been used in combination with the CHARMM36m force field [7] and has been shown to yield satisfactory results for disordered regions.

Addition to the text. (Results section: The hIRE1*α* cLD forms a stable dimer) “The TIP4P-D water model was developed to address limitations of existing force fields in reproducing the structural ensembles of intrinsically disordered proteins and regions. It incorporates enhanced dispersion and moderately stronger electrostatic interactions to improve the balance between water dispersion and electrostatics [8]. Zapletal et al. [7] showed that for proteins containing both folded and disordered regions, the CHARMM36m force field [9] in combination with the TIP4P-D water model provides a robust framework, preventing collapse of disordered regions while preserving folded regions. Acknowledging that the behavior of disordered regions can be case-specific, we conducted molecular dynamics simulations of the two cLD dimer models using the CHARMM36m force field with both TIP3P and TIP4P-D water models.”

(6) I suggest referring to the methodology part for simulation details as much as possible when presenting the story.

We thank the Reviewer for this suggestion. In the revised manuscript, we now refer the reader to the Methodology section for detailed descriptions of the HDX-MS data analysis and the MM/PBSA free energy calculations.

Addition to the text. (Results section: Hydrogen-deuterium exchange experimental data validate the cLD dimer structure) “From our simulations, we calculated the theoretical deuterated fraction using the method by Bradshaw et al.[10] and compared it to the experimental data (Fig. 1C-D and Supplementary Fig. 10) (see Methods).”

Addition to the text. (Results section: Unfolded polypeptides bind to hIRE1*α* cLD dimer surface) “We further assessed the MPZ-derived peptide complexes using MM/PBSA free energy calculations over the final 250 ns of each simulation replica (see Methods), finding binding enthalpies consistent with our observations (Supplementary Fig. 16B). In particular, MPZ1N-2X exhibited the lowest binding energy, whereas MPZ1N-2X-RD showed the highest.”

(7) Error bars and methodology of error analysis should be provided for all cases of all-atom simulations if possible, since convergence is always an issue when considering these conformational changes within microseconds of all-atom simulations.

We thank the Reviewer for the important observation. We agree and added error methodology for the estimation of theoretical deuterated fractions (Fig. 1C).

Addition to the text. (Figure C legend) “Each point represents the mean value derived from three replicas and two monomers per replica. The error bars were obtained from bootstrapping.”

Addition to the text. (Methods section: Hydrogen-deuterium exchange fractions calculation from MD simulations) “To reproduce the time points after incubation in deuterium (D_2_O), we computed deuterated fractions separately for each of the two monomers constituting a dimer for the time points 0.5 min (30 s) and 5 min (300 s). Then, we computed the mean and standard deviation over the data coming from replicas of the same cLD dimer model (AF or PDB model) and the same water model (TIP3P or TIP4P-D). To estimate the uncertainty of the mean values obtained from our datasets and the dataset from Amin-Wetzel et al. ([11] Figure 3—source data 1), we applied a non-parametric bootstrap resampling procedure. For each sequence range from HDX-MS analysis, we treated the measurements from the N=6 independent datasets as independent samples, accounting for 3 replicas each with two monomers (6 monomers total). We then generated 10,000 bootstrap replicates by sampling the datasets with replacement, maintaining the same number of samples N in each resample. For each replicate, we calculated the mean at each sequence position. The resulting distribution of bootstrap means was used to compute the standard deviation as an estimate of the standard error. We computed the difference between simulation and experimental data (deuterated fraction discrepancy), and for each residue, we selected as the ‘best structure’ the model with the discrepancy closest to zero among PDB-TIP3P, PDB-TIP4P-D, AF-TIP3P, and AF-TIP4P-D systems.”

(8) Technically I would call DR1 and DR2 linker regions within a folded structure. Their motions are quite restrained by the fold part. I therefore, am not sure how much TIP4PD really helps in contrast to a scaled TIP3P. A plot of structures colored with PLDDT score or b-factor within the PDB should be provided. Quantitative metrics of these regions (e.g. chi chi-squared) might help justify the choice of the AF model against the PDB model. Currently, the two models look very similar in Figures 1c and 1d. Similarly, quantitative metrics as a function of different simulation time windows will help justify the convergence of the simulation and indicate the flexibility of these regions.

We thank the Reviewer for this thoughtful comment. In response, we analyzed the AlphaFold2 and AlphaFold3 predictions, which consistently assign very low pLDDT values (<50) to the DR2 region, while DR1, is predicted with higher but still low confidence (50 < pLDDT < 70). These scores indicate intrinsic uncertainty in the structural definition of both regions, supporting their flexibility despite being located within a folded context.

Addition to the text. (Results section: The hIRE1_α_ cLD forms a stable dimer) “All five AlphaFold 2 predictions closely resembled the top-ranked model used for our simulations (Supplementary Fig. 7C). In contrast, the five AlphaFold 3 predictions yielded greater variability in DR2 organization and longer helices in DR2, but still consistently maintain low pLDDT scores in this region, indicating disorder (Supplementary Fig. 7D).”

Addition to the text. (Figure 7 C-D legend) “(C) Superposition of the 5 structures predicted by AlphaFold 2 Multimer for the cLD dimer and colored by confidence prediction score (pLDDT). (D) Superposition of the 5 structures predicted by AlphaFold 3 for the cLD dimer and colored by confidence prediction score (pLDDT).”

(9) Fluorescence anisotropy seems to be an important set of experimental data to justify the binding of multiple unfolded peptides to IRE. I suggest the authors include a bar plot of binding affinity of different variants in Figure 3. The raw titration curves should also be included in SI.

We thank the Reviewer for this valuable suggestion. The binding affinities reported in previous studies are summarized in Table 2; the reader is referred to those works for the corresponding raw titration curves. The binding affinities for the cLD mutants analyzed in the present study are provided in Table 3, and the associated titration curves are shown in Figure 4G.

Addition to the text. (Figure 4G legend) “Fluorescence anisotropy measurements of labeled MPZ1N-2X binding to hIRE1*α* LD wild type and mutants E102R and Y161R.”

Addition to the text. (Supplementary figures and tables: Table 3) See Tab. 1

(10) The authors should discuss the dependence of initial orientations of unfolded peptides on the final results. The authors claimed that after 1 microsecond simulations, the orientation of these peptides to IRE changed. Quantitative metrics showing both the binding (e.g., number of contacts) and binding orientation (contact region or angles) should be provided to tell whether the simulation is converged. The comparison to the experimental data lacks quantitative metrics. The authors mentioned the dissociation of MPZ1N-2X-RD in half of the simulations; they might want to provide such a metric for all peptides. Technically, 1 microsecond brute-force simulation is quite short for observing such a binding event, and enhanced sampling methods (e.g. metadynamics) might be necessary for investigating binding. However, at least the presentation and interpretation of the current results should be improved for comparing simulations and experiments.

We thank the Reviewer for the insight. We expanded the discussion of the peptide orientation and added an analysis of the peptide angle with respect to the cLD central groove and contacts. Additionally, we inserted AlphaFold 3 predictions of all the simulated complexes.

Addition to the text. (Results section: Unfolded polypeptides bind to hIRE1_α_ cLD dimer surface) “In initial simulations with peptides valine8 and MPZ1-N, we positioned the polypeptides over the cLD, aligning them parallel to the principal axis of the central groove in accordance with the proposed binding mode. We refer to this pose as the "0◦ orientation", as the peptide forms a 0 ◦ angle with the principal axis of the groove. We observed that the peptides could rearrange into an orientation perpendicular to the central groove axis, while maintaining contact with the dimer (Fig. 3A, Supplementary Fig. 13A, valine8 TIP4P-D, and Supplementary Fig. 14). Conversely, when MPZ1-N was initially oriented perpendicularly to the groove, it did not transition to a parallel (0◦) orientation (Supplementary Fig. 14). We refer to these poses as the "90◦ orientation" and "270◦ orientation".”

Addition to the text. (Supplementary Figures and Tables Fig. 14) “(A) Peptide orientation with respect to the central groove principal axis. The angle was computed as the dihedral angle described by the C*α* atoms of Y161 residues (groove principal axis) and the C_α_ atoms of residues L1 and A12 of the MPZ1N peptide. The dark lines indicate the rolling average of the fraction of native contacts over 10 frames, while the shaded lines indicate the value per frame. (B) Number of contacts between hIRE1*α* cLD dimer and MPZ1N peptide. The dark lines indicate the rolling average of the fraction of native contacts over 50 frames, while the shaded lines indicate the value per frame. The analysis were performed on three sets of simulations: "90 degrees" orientation, the peptide is initially placed perpendicular to the central groove principal axis; "270 degrees" orientation, the peptide is initially placed perpendicular to the central groove principal axis but flipped 180 degrees with respect to the 0 degree; "0 degrees" orientation, the peptide is placed parallel to the groove principal axis.”

Addition to the text. (Results section: Unfolded polypeptides bind to hIRE1*α* cLD dimer surface) “AlphaFold3 predictions of the complexes indicate that the peptides adopt the same preferred orientation, despite being predominantly helical (Supplementary Fig. ??A).”

Addition to the text. (Supplementary Figures and Tables Fig. 16A) “(A) Prediction of AlphaFold 3 for hIRE1*α* cLD dimer in complex with peptides. Colors represent the confidence of the prediction (plDDT).”

(11) I also have a couple of questions regarding the point mutant Y161R. (a) The motivation of mutating Y161 to R is more speculative (Figures 4a,b) than quantitative. The authors might want to show an intermolecular contact map between IRE and unfolded peptides or IRE contact probability along residue indexes to show the interaction hotspots. Figure S11 only showed the structure instead of any metrics for such a purpose. (b) It might be better to also show a histogram of the distances of Figure 4e and 4f. Figure 4f actually suggested 1 microsecond simulation is quite short to observe the dissociation event. (c) Testing the mutation within the experiment, if possible, would clearly strengthen this part of the manuscript.

We thank the Reviewer for these constructive suggestions. We have added an analysis of intermolecular contacts for the Y161R and E102R mutants (Fig. 18A–B), which highlights the interaction hotspots between IRE1 residues and the unfolded peptides. To further characterize peptide–groove interactions, we now provide minimum peptide–groove distance time series for all peptides (Fig. 15B). Moreover, to experimentally support our simulations, we performed fluorescence anisotropy measurements on the MPZ1N-2X peptide with cLD WT and mutant constructs. These experiments confirm our computational observations (Fig. 4F–G and Fig. 18C).

Addition to the text. (Figure 18 legend) “(A) Number of contacts between residues 102 on both monomers and the MPZ1-N-2X peptide during simulations of WT hIRE*α* LD and mutants E10R and Y161R. The dark lines indicate the rolling average of the fraction of native contacts over 25 frames, while the shaded lines indicate the value per frame. (B) Number of contacts between residues 161 on both monomers and the MPZ1-N-2X peptide during simulations of WT hIRE*α* LD and mutants E10R and Y161R. The dark lines indicate the rolling average of the fraction of native contacts over 25 frames, while the shaded lines indicate the value per frame. (C) Protein purification of WT hIRE*α* LD and mutants E10R and Y161R.”

Addition to the text. (Figure 4F-G legend) “(F) Time series of the minimum groove-peptide distance for MPZ1N-2X simulated in complex with wild-type, E102R, and Y161R hIRE1*α* cLD dimer in TIP3P (3 replicas) and TIP4P-D (3 replicas) water. The darker lines show the rolling average over 25 frames, while the shaded lines represent the raw data. (G) Fluorescence anisotropy measurements of labeled MPZ1N-2X binding to hIRE1*α* LD wild type and mutants E102R and Y161R.”

Addition to the text. (Figure 15B legend) “(B) Minimum groove-peptide distance over time for all simulations of cLD dimer in complex with a peptide. The left column shows the values for the three replicas in TIP3P water, while the right column displays those for the three replicas in TIP4P-D water.”

(12) Similar comments of quantitative analysis (e.g. contact map as a function of simulation time) apply to the last part of results when discussing the intermolecular interactions. Observations such as "the interface predicted by AlphaFold showed stability across MD simulation replicas lasting 200 ns" were provided, but there is no quantitative analysis. How consistent was this observation across multiple replicas of simulations, and how many replicas were used?

We thank the Reviewer for this valuable suggestion. To provide a quantitative assessment, we performed new triplicate simulations of the BiP–cLD monomer complex and plotted the fraction of native contacts over time. These results, which demonstrate the consistency of the interface across replicas, are now included in the Supplementary Material.

Addition to the text. (Figure 19 legend) “(A) Prediction of AlphaFold 3 for hIRE1*α* cLD monomer in complex with ATP-bound BiP. The colors are as in Fig. 5B. (B) Prediction of AlphaFold 3 for hIRE1α cLD monomer in complex with ADP-bound BiP. (C) Prediction of AlphaFold 3 for hIRE1*α* cLD monomer in complex with BiP not bound to any nucleotide. (D) Structure of hIRE1α cLDBiP-ATP after 2 µs of simulation. (E) Structure of hIRE1*α* cLD-BiP-ADP after 2 µs of simulation. (F) Structure of hIRE1*α* cLD-BiP after 2 µs of simulation.”

Addition to the text. (Figure 20 legend) “Fraction of native contacts between BiP and cLD monomer in simulations of the structures predicted by AlphaFold 3 without ligands or in complex with ADP or ATP. The dark lines indicate the rolling average of the fraction of native contacts over 100 frames, while the shaded lines indicate the value per frame. The fraction of native contacts (Q) was calculated according to the definition of Best et al. [12]: $Q(X)=\frac{1}{N} \sum_{(i, j)} \frac{1}{1+\exp \left[\beta\left(r_{(i, j)}(X)-\lambda r_{(i, j)}^{0}\right) \right]}$. For *N* pairs of native contacts (*i, j*), where $r_{(i, j)}^{0}$ is the distance of the pair in the initial configuration (here the AlphaFold 3 prediction), *r_(i,j)_*(*X*) is the distance at frame X, *β* is a smoothing parameter (*β* = 50 *nm*^−1^), *λ* is the tolerance of the reference distance (*λ* \= 1.8) and the cutoff used to define a contact between heavy atoms was 0.45 nm.”

(13) The figure legends are noted using lowercase letters but are described using uppercase.

We thank the Reviewer for pointing that out, and we changed everything to capital letters.

**Reviewer #3 (Recommendations for the authors):**

(1) Figure 1: I am confused about the HDX-MS results shown in Figure 1. Here, I must also mention that I am not familiar with comparing HDX-MS experiments with MD simulations. The authors mention that they show the deuterated fraction computed from MD simulations for the PDB and AF model at time points 0.5 min and 5 min. However, this time certainly does not correspond to the MD simulation time, thus, it is unclear to me where the difference between the results comes from. Are the two time points some input parameters to the script used to calculate the deuterated fraction? Thus, I would ask the authors to better explain what is the difference in the results between the two time points. Especially, since the general reader might not be familiar with comparing HDX-MS experimental results to MD simulations. Furthermore, I would ask the authors to clarify in the Figure 1 caption that these time points do not correspond to the MD simulation time.

We thank the Reviewer for pointing us to this possible source of confusion. The time points are effectively input parameters to the calculations of theoretical deuterated fractions from MD simulations. We expanded the explanation of the method in the method section and clarified in the Figure 1 caption that these time points do not correspond to the MD simulation time.

Addition to the text. (Methods section: Hydrogen-deuterium exchange fractions calculation from MD simulations) “To determine the deuterated fraction of a peptide segment from simulations, the protection factor for each residue *i*, *Pi*, must be computed from the simulation snapshots, following the approach of Best and Vendruscolo [13]: $\ln P_{i}=\left\langle\beta_{C} N_{C, i}+\beta_{H} N_{H, i}\right\rangle$. Here, *N_C,i_* and *N_H,i_* are the number of H-bonds and heavy-atom contacts of the backbone amide of residue *i*, and the scaling factors *β_C_* and *β_H_* are set to 0.35 and 2.0, respectively. The simulated deuterated fraction of a peptide segment, $D_{i, j}^{\operatorname{sim}}$, defined by residues *m_j_* +1 to *n_j_*, was then calculated at any exchange time point *t* as:

$D_{i, j}^{\mathrm{sim}}=\frac{\sum_{i=m_{j}+1}^{n_{j}} 1-\exp \left(-\frac{k^{\mathrm{lint}}}{P_{i}} t\right)}{n_{j}-m_{j}}$

Where *m_j_* and *n_j_* are the first and last residue numbers of the j-th protein fragment, respectively. The intrinsic exchange rate constants for each residue type ($k_{i}^{\mathrm{int}}$) were obtained from Bai et al. with updated acidic residues and glycine [14, 15].”

Addition to the text. (Figure 1 legend:) “This time point corresponds to experimental incubation times, not MD simulation time.”

Addition to the text. (Figure 10 legend:) “Time points correspond to experimental incubation times, not MD simulation time.”

(2) For AlphaFold 2 Multimer prediction, the authors only considered the top predicted structure. However, AF2-M, one generally obtains 5 structures, and it is also possible to obtain more structures by using an additional random seed. Thus, it would be interesting if the authors would consider the difference between the 5 structures they obtained from the AF2-M prediction. Are they all very similar? (Especially considering the DR1 and DR2 segments, that is the main difference between the PDB and AF2 structures). Analyzing the different predicted AF2 structures would give more insight into the accuracy of the AF2-M predicted model.

We thank the Reviewer for this insightful suggestion. All AF2-M predicted structures were found to be highly similar, and we now include them in Figure 7E for comparison.

Addition to the text. (Figure 7E legend) “(E) Superposition of the 5 structures predicted by AlphaFold 2 Multimer for the cLD dimer and colored by confidence prediction score (pLDDT).”

(3) On Page 6, the authors talk about a "an early PDB model". First, I find the nomenclature "early" confusing here; perhaps it would be better to talk about "an initial PDB model", but I leave it up to the authors to think about if they want to change that. More importantly, reading the Comp. detail on Page 23, it is not so clear what the difference is between the "early" and "final" PDB models, and how the difference in their setups leads to different results. The information is somewhat there on Page 6 and Page 23, but it can be made much clearer. Thus, I would ask the authors to better explain the difference between the early and final PDB models.

We thank the Reviewer for this helpful comment. In the revised manuscript, we have clarified the terminology and provided a more explicit explanation of the differences between the two IRE1 models, both in the Results section and in the Methods.

Addition to the text. (Results section: The hIRE1*α* cLD forms a stable dimer) “An initial PDB model with modified side chain orientations in residues L116 and Y166 due to the modelling of neighbouring missing DR1, caused the dimer to dissociate in one-third of the replicas. [...] The final PDB model, with correctly oriented L116 and Y166 (Supplementary Fig. 9B), was stable in simulations in both TIP3P and TIP4P-D water (Supplementary Fig. 7B).”

Addition to the text. (Methods section: IRE1_α_ core Luminal Domain (cLD) structural models - Human PDB dimer) “An initial PDB model was briefly equilibrated in NPT, and a conformation with a groove width of approximately 0.6 nm was selected. This snapshot was used as the initial structure for the initial “PDB model” simulations, in which the dimer dissociates.”

(4) Page 12: "In early simulations", again, I find the nomenclature "early" confusing here. Perhaps it would be better to talk about "In initial simulations" or "In preliminary simulations", but I leave it up to authors to think about this.

We thank the Reviewer for pointing out this possible source of confusion. We improved the text by referring to these simulations based on the different orientations of the peptide on the cLD dimer in the modeled complex.

Addition to the text. (Results section: Unfolded polypeptides bind to hIRE1_α_ cLD dimer surface) “In initial simulations with peptides valine8 and MPZ1-N, we positioned the polypeptides over the cLD, aligning them parallel to the principal axis of the central groove in accordance with the proposed binding mode. We refer to this pose as the "0° orientation", as the peptide forms a 0° angle with the principal axis of the groove. We observed that the peptides could rearrange into an orientation perpendicular to the central groove axis, while maintaining contact with the dimer (Fig. 3A, Supplementary Fig. 13A, valine8 TIP4P-D, and Supplementary Fig. 14). Conversely, when MPZ1-N was initially oriented perpendicularly to the groove, it did not transition to a parallel (0°) orientation (Supplementary Fig. 14). We refer to these poses as the "90° orientation" and "270° orientation".”

Here, we provide a detailed description of the additional changes made to the manuscript.

Additional edits to the manuscript

Following discussions with Prof. Dr. David Ron, we refined our BiP model by removing the signal peptide (residues 1–18). Using AlphaFold 3, we predicted BiP–cLD heterodimeric complexes in the presence of ADP, ATP, or without nucleotide. Each of the three complexes was simulated in TIP3P water, in three independent replicas of 1 µs each.

Addition to the text. (Results section: hIRE1*α* cLD intermolecular interactions guide the activation process) “We used AlphaFold 3 to model the interaction between a cLD monomer and BiP (residues E19–L654) in the presence of ATP and ADP (Fig. 5B, Supplementary Fig. 19A). Prediction quality was limited in the apo and ADP-bound states (pTM = 0.48, ipTM = 0.59; pTM = 0.49, ipTM = 0.61, respectively), whereas ATP binding improved accuracy (pTM = 0.66, ipTM = 0.72). The predicted interfaces involved DR2, particularly residues 314PLLEG-318, forming a short parallel *β*-sheet with the substrate-binding domain (SBD) of BiP through two hydrogen bonds. All AlphaFold 3 models were stable across three 1-µs simulations (Supplementary Fig. 19B), with cLD–BiP interfaces retaining 60–80% of initial contacts (Supplementary Fig. 20). In the apo and ADP-bound states, the nucleotide-binding domain (NBD) showed high Predicted Aligned Error (PAE) relative to the cLD, indicating uncertain positioning of the two domains relative to each other. Notably, in the ADP-bound state, which is thought to interact with hIRE1*α* cLD, the NBD remained mobile but proximal to the *α*B-helices, thereby restricting access to this region. Together, the AlphaFold 3 predictions suggest that BiP engages hIRE1*α* cLD by sterically hindering the oligomerization interface defined by DR2 and the αB-helices [16].”

Addition to the text. (Figure 5 legend) “(B) BiP-cLD monomer complex as predicted by AlphaFold (BiP in shades of purple, cLD in orange) before the simulation (t = 0 *µ*s) and at the end of the simulation (t = 1 *µ*s). The SBD (residues E19-D408) is colored in light purple, and the NDB (residues C420-E650) in dark purple, and the interdomain linker (residues D409-V419) and KDEL motif (residues K651-L654) in light purple.”

Addition to the text. (Figure 19 legend) “(A) Prediction of AlphaFold 3 for hIRE1α cLD monomer in complex with ATP-bound BiP. The colors are as in Fig. 5B. (B) Prediction of AlphaFold 3 for hIRE1*α* cLD monomer in complex with ADP-bound BiP. (C) Prediction of AlphaFold 3 for hIRE1*α* cLD monomer in complex with BiP not bound to any nucleotide. (D) Structure of hIRE1*α* cLDBiP-ATP after 2 µs of simulation. (E) Structure of hIRE1*α* cLD-BiP-ADP after 2 µs of simulation. (F) Structure of hIRE1*α* cLD-BiP after 2 µs of simulation.”

Addition to the text. (Methods section: cLD monomer in complex with BiP) “The BiP-cLD heterodimer systems were predicted with AlphaFold 3 using the AlphaFold server[17] at https://alphafoldserver.com/. The hIRE1*α* cLD sequence used is the same used for predicting the dimer: the PDB 2HZ6 sequence, Uniprot identifier O75460 with mutations C127S and C311S, and residues P29-P368. The BiP sequence used is taken from UniProt identifier P11021, residues E19L654. We predicted three complexes: one without any nucleotide, one containing ADP, and another containing ATP. Simulations of the BiP-cLD complex were run in TIP3P water.”

We have updated the Zenodo repository with additional data and calculations, and the corresponding link is provided in the manuscript.

References

(1) Mario S. Valdés-Tresanco, Mario E. Valdés-Tresanco, Pedro A. Valiente, and Ernesto Moreno. gmx_mmpbsa: A New Tool to Perform End-State Free Energy Calculations with GROMACS. Journal of Chemical Theory and Computation, 17(10):6281–6291, October 2021. Publisher: American Chemical Society.

(2) Bill R. III Miller, T. Dwight Jr. McGee, Jason M. Swails, Nadine Homeyer, Holger Gohlke, and Adrian E. Roitberg. MMPBSA.py: An Efficient Program for End-State Free Energy Calculations. Journal of Chemical Theory and Computation, 8(9):3314–3321, September 2012. Publisher: American Chemical Society.

(3) Fanhao Wang, Yuzhe Wang, Laiyi Feng, Changsheng Zhang, and Luhua Lai. Target-Specific De Novo Peptide Binder Design with DiffPepBuilder. Journal of Chemical Information and Modeling, 64(24):9135–9149, December 2024. Publisher: American Chemical Society.

(4) Alexander D. MacKerell Jr., Bernard Brooks, Charles L. Brooks III, Lennart Nilsson, Benoit Roux, Youngdo Won, and Martin Karplus. CHARMM: The Energy Function and Its Parameterization. In Encyclopedia of Computational Chemistry. 2002.

(5) Bernard R. Brooks, Robert E. Bruccoleri, Barry D. Olafson, David J. States, S. Swaminathan, and Martin Karplus. CHARMM: A program for macromolecular energy, minimization, and dynamics calculations. Journal of Computational Chemistry, 4(2):187–217, 1983.

(6) Junxi Mu, Hao Liu, Jian Zhang, Ray Luo, and Hai-Feng Chen. Recent Force Field Strategies for Intrinsically Disordered Proteins. Journal of Chemical Information and Modeling, 61(3):1037–1047, March 2021.

(7) Vojtech Zapletal, Arnošt Mládek, Kateˇ ˇrina Melková, Petr Louša, Erik Nomilner, Zuzana Jasenáková, Vojtˇ ech Kubáˇ n, Markéta Makovická, Alice Laníková, Lukášˇ Žídek, and Jozef Hritz. Choice of Force Field for Proteins Containing Structured and Intrinsically Disordered Regions. Biophysical Journal, 118(7):1621–1633, April 2020.

(8) Stefano Piana, Alexander G. Donchev, Paul Robustelli, and David E. Shaw. Water dispersion interactions strongly influence simulated structural properties of disordered protein states. Journal of Physical Chemistry B, 119(16):5113–5123, April 2015.

(9) Jing Huang, Sarah Rauscher, Grzegorz Nawrocki, Ting Ran, Michael Feig, Bert L. de Groot, Helmut Grubmüller, and Alexander D. MacKerell. CHARMM36m: an improved force field for folded and intrinsically disordered proteins. Nature Methods, 14(1):71–73, January 2017.

(10) Richard T. Bradshaw, Fabrizio Marinelli, José D. Faraldo-Gómez, and Lucy R. Forrest. Interpretation of HDX Data by Maximum-Entropy Reweighting of Simulated Structural Ensembles. Biophysical Journal, 118(7):1649–1664, April 2020.

(11) Niko Amin-Wetzel, Lisa Neidhardt, Yahui Yan, Matthias P. Mayer, and David Ron. Unstructured regions in IRE1 specify BiP-mediated destabilisation of the luminal domain dimer and repression of the UPR. eLife, 8, December 2019.

(12) Robert B. Best, Gerhard Hummer, and William A. Eaton. Native contacts determine protein folding mechanisms in atomistic simulations. Proceedings of the National Academy of Sciences, 110(44):17874–17879, October 2013. Publisher: Proceedings of the National Academy of Sciences.

(13) Robert B. Best and Michele Vendruscolo. Structural Interpretation of Hydrogen Exchange Protection Factors in Proteins: Characterization of the Native State Fluctuations of CI2. Structure, 14(1):97–106, January 2006.

(14) Yawen Bai, John S. Milne, Leland Mayne, and S. Walter Englander. Primary structure effects on peptide group hydrogen exchange. Proteins: Structure, Function, and Bioinformatics, 17(1):75–86, 1993. _eprint: here.

(15) David Nguyen, Leland Mayne, Michael C. Phillips, and S. Walter Englander. Reference Parameters for Protein Hydrogen Exchange Rates. Journal of the American Society for Mass Spectrometry, 29(9):1936–1939, September 2018. Publisher: American Society for Mass Spectrometry. Published by the American Chemical Society. All rights reserved.

(16) G Elif Karagöz, Diego Acosta-Alvear, Hieu T Nguyen, Crystal P Lee, Feixia Chu, and Peter Walter. An unfolded protein-induced conformational switch activates mammalian IRE1. eLife, 6:e30700, 2017.

(17) Josh Abramson, Jonas Adler, Jack Dunger, Richard Evans, Tim Green, Alexander Pritzel, Olaf Ronneberger, Lindsay Willmore, Andrew J. Ballard, Joshua Bambrick, Sebastian W. Bodenstein, David A. Evans, Chia-Chun Hung, Michael O’Neill, David Reiman, Kathryn Tunyasuvunakool, Zachary Wu, Akvile Žemgu-˙ lyte, Eirini Arvaniti, Charles Beattie, Ottavia Bertolli, Alex Bridgland, Alexey˙ Cherepanov, Miles Congreve, Alexander I. Cowen-Rivers, Andrew Cowie, Michael Figurnov, Fabian B. Fuchs, Hannah Gladman, Rishub Jain, Yousuf A. Khan, Caroline M. R. Low, Kuba Perlin, Anna Potapenko, Pascal Savy, Sukhdeep Singh, Adrian Stecula, Ashok Thillaisundaram, Catherine Tong, Sergei Yakneen, Ellen D. Zhong, Michal Zielinski, Augustin Žídek, Victor Bapst, Pushmeet Kohli, Max Jaderberg, Demis Hassabis, and John M. Jumper. Accurate structure prediction of biomolecular interactions with AlphaFold 3. Nature, pages 1–3, May 2024.
